# Study on the Effect of Three Types of Calcium Sulfate on the Early Hydration and Workability of Self-Compacting Repair Mortar

**DOI:** 10.3390/ma16165648

**Published:** 2023-08-16

**Authors:** Hao Ding, Xuepeng Shen, Aili Chen, Rulin Gu, Ying Fang, Dongxu Li

**Affiliations:** 1 College of Materials Science and Engineering, Nanjing Tech University, Nanjing 210009, China; 202161203249@njtech.edu.cn (H.D.);; 2 Nanjing Kunchang New Material Co., Ltd., Nanjing 210000, China; 3 College of Materials Science and Engineering, Zhejiang University, Hangzhou 310058, China

**Keywords:** repair mortar, calcium sulfate, ettringite, cement

## Abstract

Despite having a high early mechanical strength and using sulfoaluminate cement as the primary cementitious material, self-compacting repair mortar (SCRM) suffers from rapid hydration rates leading to construction time constraints. This study examined how several forms of calcium sulfate, including hemihydrate gypsum, anhydrite, and dihydrate gypsum, affected SCRM’s workability, hydration process, and microstructure. The outcomes demonstrated that adding hemihydrate gypsum sped up SCRM’s early hydration rate and boosted its expansion rate. For a cement with 8% hemihydrate gypsum, 6 h after adding the water, the flexural strength and compressive strength increased by 39.02% and 34.08%, respectively. The hydration rate of SCRM can be efficiently delayed by dihydrate gypsum, although the result is subpar. The material exhibited the lowest fluidity loss in 20 min, the setting time was extended, and the 28-day flexural and compressive strengths were raised by 26.56% and 28.08%, respectively, after adding 8 percent anhydrite.

## 1. Introduction

When used to repair construction and building structures, self-compacting repair mortar (SCRM) is a cement mortar made up of cement, mineral admixtures, fine aggregates, additives, etc., in the proper proportions. SCRM requires stirring with a specific amount of water or other liquids [1,2,3,4,5]. SCRM should also possess qualities including a suitable setting time, increased fluidity, stronger early strength, good resistance to segregation, and stable volume change in order to match the building criteria [6]. When silicate cement (PC) is utilized as the primary cementitious material for SCRM, SCRM would typically shrink and crack due to issues with PC’s poor hydration rate, low early strength, and extreme drying shrinkage [7]. Sulfoaluminate cement (SAC) is distinguished by exceptional properties including early strength, high strength, frost resistance, impermeability, corrosion resistance, and low alkalinity [8,9,10]. This type of cement also exhibits a very promising future due to its quick setting time rate [11,12,13,14]. The C_4_A_3_ minerals in SAC and the C_3_S minerals in PC have a mutually encouraging impact on the common hydration process when sulfoaluminate cement and silicate cement are combined, hastening the hydration and setting of the combined cement [15]. Calcite alumina (AFt), aluminum gel, C-S-H gel, and a minor amount of low-sulfur hydrated calcium sulfoaluminate are the major hydration products of this composite system [16,17,18]. The early strength of the system is provided by the creation of AFt, while the development of C-S-H gels ensures the later increase in cement strength [13,19].

As was previously noted, the primary hydration reactant in sulfoaluminate cements is sulfoaluminate. During the hydration process, calcium sulphate is typically used, and the following reactions result in the creation of AFt (C_6_A$\overset{\text{\_}}{S}$_3_H_32_) and aluminum hydroxide (AH_3_):

(1) $$C_{4}A_{3}\overset{\text{\_}}{S}+2C\overset{\text{\_}}{S}+38H_{2}O\to C_{6}A{\overset{\text{\_}}{S}}_{3}H_{32}+2AH_{3}$$

At low calcium sulfate concentrations, anhydrous calcium sulfoaluminate can also be hydrated:

(2) $$C_{4}A_{3}\overset{\text{\_}}{S}+20H_{2}O\to C_{4}A\overset{\text{\_}}{S}H_{14}+2AH_{3}$$

In the presence of a high alumina phase, the silicate component goes through the following hydration processes to produce [20]:

(3) $$C_{2}S+AH_{3}+5H_{2}O\to C_{2}ASH_{8}$$

Strätlingite (C_2_ASH_8_), which is also known as the AFm phase [21,22], is formed when C_2_S is present and may have a reduced crystallinity because of the existence of vacancy sites and partial occupancy of specific groups (e.g., hydroxyl groups and water molecules).

To enhance the early performance of silicate cements, researchers have occasionally tried mixing sulfoaluminate cements with regular silicate cements. In this mixed system, a variety of reactions take place, resulting in the creation of AFt:

(4) $$C_{4}A_{3}\overset{\text{\_}}{S}+8C\overset{\text{\_}}{S}+6CH+90H_{2}O\to 3C_{6}A{\overset{\text{\_}}{S}}_{3}H_{32}$$

Studies on the hydration of sulfoaluminate cements have shown that the creation of AFt is crucial for cementitious materials’ setting and final characteristics [10]. In instance, the AFt created in Reaction (1) exhibits remarkable dimensional stability [23], in contrast to the swelling AFt created in Reaction (4). The formation mechanism and microstructural characteristics of AFt vary depending on the calcium sulfate’s source [6,24,25]. When hard gypsum is employed, sluggish release of sulfate ions impacts the kinetics of AFt formation, but on the other hand, results in the development of AFt with tight lattice structure [26,27,28]. Rapid gypsum dissolution speeds up the formation of AFt and creates a thick matrix on the basis of tiny crystals.

Fewer studies have been performed on the impact of various kinds of calcium sulfate on repair mortars despite the fact that there are many researchers studying the hydration mechanism and material characteristics of the ternary cementitious system of sulfoaluminate cement, common silicate cement, and gypsum. In this study, three different types of calcium sulfate, α-hemihydrate gypsum, anhydrite, and dihydrate gypsum, were used for the preparation of SCRM. The effects of calcium sulfate on the early hydration and properties of SCRM were investigated. Specifically, their effects on the working properties of SCRM, such as mechanical strength, setting time, and fluidity, were studied. The hydration process and hydration products were investigated with X-ray diffractometry (XRD) and isothermal heat conduction calorimetry, and the microstructures were investigated with the mercuric pressure method (MIP). The mechanism of different types of calcium sulfate was explored to provide theoretical basis for the preparation of ternary repair mortar.

## 2. Materials and Methods

### 2.1. Materials

**Figure 1:**
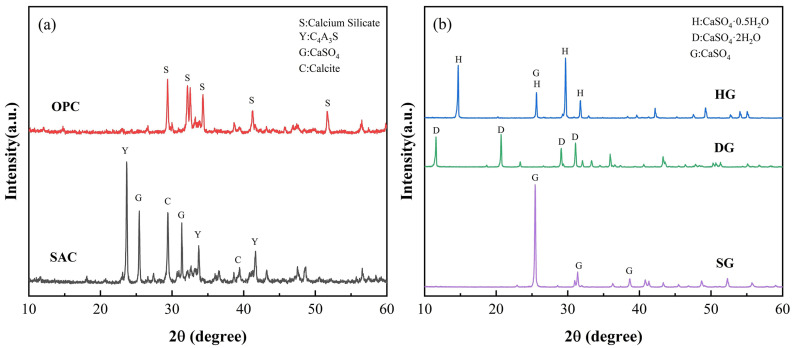
X-ray diffraction patterns of raw materials. (**a**) Diffraction pattern of cement; (**b**) diffraction pattern of calcium sulfate.

SAC used low-alkalinity sulfoaluminate cement (L-SAC 42.5) produced by Zhengzhou Cuanzhu New Material Technology Co., Ltd. (Zhengzhou, China); silicate cement used ordinary silicate cement (P.O 42.5R) produced by Anhui Conch Cement Company Limited. The specific surface areas of α-hemihydrate gypsum (HG), anhydrite (SG), dihydrate gypsum (DG) were 364 m^2^/kg, 639 m^2^/kg, and 521 m^2^/kg, respectively; the chemical compositions of the cement and calcium sulfate determined by X-ray fluorescence (XRF) are shown in Table 1. Figure 1 shows the phase compositions of cement and calcium sulfate determined by X-ray diffraction (XRD). Retarder—tartaric acid, water reducing agent—polycarboxylic acid high-efficiency water reducing agent (1802B), cellulose—carboxycellulose methyl ether (relative molecular weight of 400), glue powder—re-dispersible emulsion powder EVA (Ethylene-EVA ethylene vinyl acetate copolymer), all produced by Nanjing Kunchang New Material Co., Ltd. (Nanjing, China); defoamer—9010F, produced by BASF; aggregate—fine river sand (mesh: 70–140); mixing water—tap water.

### 2.2. Test Methods

#### 2.2.1. Test Formulations

This test ternary system is formulated as shown in Table 2 using gypsum and other mass replacement cement; the dosage is 4 wt% and 8 wt%, the water–cement ratio is 0.37, the glue–sand ratio is 1:1.5, the water reducer dosage is 0.2 wt%, the defoamer dosage is 0.1 wt%, the cellulose dosage is 0.03 wt%, the retardant dosage is 0.05 wt%, and the dosage of gum powder is 1 wt%.

#### 2.2.2. Sample Preparation

**Figure 2:**
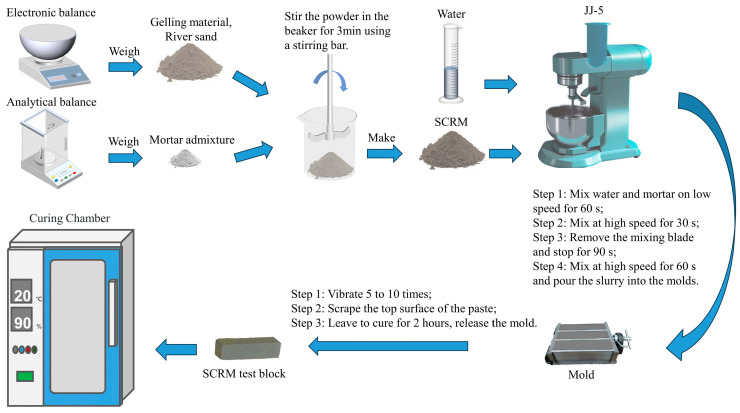
Preparation of self-compacting repair mortar (SCRM) test flowchart.

As shown in Figure 2, in an experiment to prepare mortar in accordance with JC/T 2381-2016 [29] “Repair Mortar,” each raw material, aggregate, and admixture was weighed and pre-mixed in accordance with the ratios in Table 2. Dry powder mortar was created by mixing dry powder with mixing water for two minutes to create a homogeneous slurry, and the slurry was then poured into a 40 × 40 × 160 mm mold. The slurry’s top was scraped after the mold was vibrated 5 to 10 times. After 2 h of laboratory curing, the molds were finally demolded and moved to a 20 °C incubator for maintenance. By allowing them to mature to the suitable age, sample specimens were created. Cement slurry samples were evaluated once they had achieved the appropriate age and undergone characterization tests like XRD and heat of hydration tests.

### 2.3. Testing Methods

The setting time is determined according to the experimental method stipulated in GB/T 1346-2001 [30], and the flow rate is determined according to the experimental method in JC/T 985-2005 [31] for the initial flow rate and 20-min flow rate. The mechanical strength of the specimen was determined according to GB/T 17671-1999 [32] “Test Method for Strength of Cement Sand (ISO Method)”, and the test was prepared and maintained until the age of 2.4 kN/s loading rate. The flexural strength and compressive strength were tested by using an automatic compressive strength tester (AEC-201, Wuxi Alicon Instrument Co., Ltd., Wuxi, China).

The dry shrinkage rate was determined according to the standard JC/T 985-2005. The SCRM fresh mortar was the poured into the mold of size 40 × 40 × 160 mm with a shrinkage probe and cured at 22 ± 1 °C and 95% RH (relative humidity). The initial length of the specimens was recorded after demolding, and then the change in length of the specimens at different ages (1 d, 7 d, 14 d, 21 d, and 28 d) was recorded. The rate of dimensional change was calculated as follows:

(5) $$Ɛ=(L_{t}-L_{0})/160\times 100\%$$

Here, in Equation (5), is the rate of dimensional change (%); L_0_ is the specimen’s starting test value (mm), L_t_ is the specimen’s test value at a specific curing age (mm), and 160 is the mortar specimen’s effective length (mm).

X-ray diffractometer (SmartlabTM 3 kW) from Rigaku, Japan was used to test the physical phase structure of the samples, with a scanning rate of 10°/min and a scanning range of 10° to 80°. The heat of hydration was measured using a TAM AIR 8 calorimeter to test the exothermic behavior of the early hydration of the samples. The mixing method was internal mixing, and the test duration was 24 h. By removing the samples’ exterior surfaces, breaking them into 2.5–4 mm pieces, and soaking them in alcohol, the pore structure was examined. They were removed and dried before the test, and the pore structure of the specimens was tested using a Quantum chrome Mercury in Pressure (MIP) instrument manufactured by Poremaster GT-6.0, Boynton Beach, FL, USA.

## 3. Results and Discussion

### 3.1. Fluidity and Setting Time

The results of the effect of three types of gypsum on the setting time and fluidity of SCRM are shown in Table 3. It can be seen from Table 3 that with the incorporation of calcium sulfate, the initial setting time and the final setting time of SCRM are significantly prolonged, which indicates that all three types of calcium sulfate have a retardation effect on SCRM. The initial setting time and final setting time of SG are the highest, which can reach 71 min and 114 min, respectively, which indicates that the effect of SG is higher than that of other types of calcium sulfate. The initial coagulation time of the experimental group with the addition of HG and DG was in the range of 40 min to 49 min, and the final coagulation time was in the range of 53 min to 62 min.

Compared with the control group, the mobility of the experimental group changed, with the initial mobility and 20 min mobility being lower than those of the blank group. The initial mobility and the mobility of the experimental group changed as compared to the control group, with the initial mobility and 20 min mobility being lower than those of the blank group. HG02 had the lowest initial and 20 min mobility, measuring 290 mm and 225 mm, respectively. With 301 mm and 271 mm, respectively, in its initial mobility and 20 min mobility, SG02 had the maximum mobility among the experimental groups. In terms of mobility loss, SG01 and SG02 saw mobility losses of 33 mm and 30 mm, respectively, less than the control group. With an increase in SG dosage, the mobility loss was minimized. The flow loss also decreased as SG dosage was increased. The maximum flow loss was recorded by HG02 at 65 mm, and HG01’s flow loss was similarly higher than that of the other groups at 61 mm, demonstrating that HG had a bigger impact on the flow loss of SCRM.

This ternary gelling system produces a significant quantity of AFt during the hydration process, and calcium sulfate with various solubilities also contributes significantly to AFt creation. The three different types of calcium sulfate used in this study have the following dissolution rate relationships: HG > DG > SG; HG’s faster dissolution rate can provide a higher concentration of Ca^2+^ and SO_4_^2−^ ions for the generation of AFt during the early hydration of the cement, whereas SG and DG’s slower dissolution rates cause less AFt to be formed. In the experimental group including HG, the quick development of AFt on the surface of unhydrated cement particles constrained the hydration of C_3_S [33], extending the SCRM’s setting time, while the quantity of AFt generated early on led to a greater loss of flowability. For the experimental group containing DG, the water molecules in the DG crystals reacted with the cement’s silicate hydrates to form a C-S-H gel. This reaction used up some of the cement’s water and decreased the fluidity of the cement paste, which led to a longer SCRM setting time with a greater loss of fluidity [34]. Finally, because SG dissolves at a slower pace than the other two in the experimental group, less AFt is produced early on. Due to the increased particle spacing and relatively significant exposure to free water, the SCRM condensation time is delayed.

### 3.2. Flexural Strength and Compressive Strength

**Figure 3:**
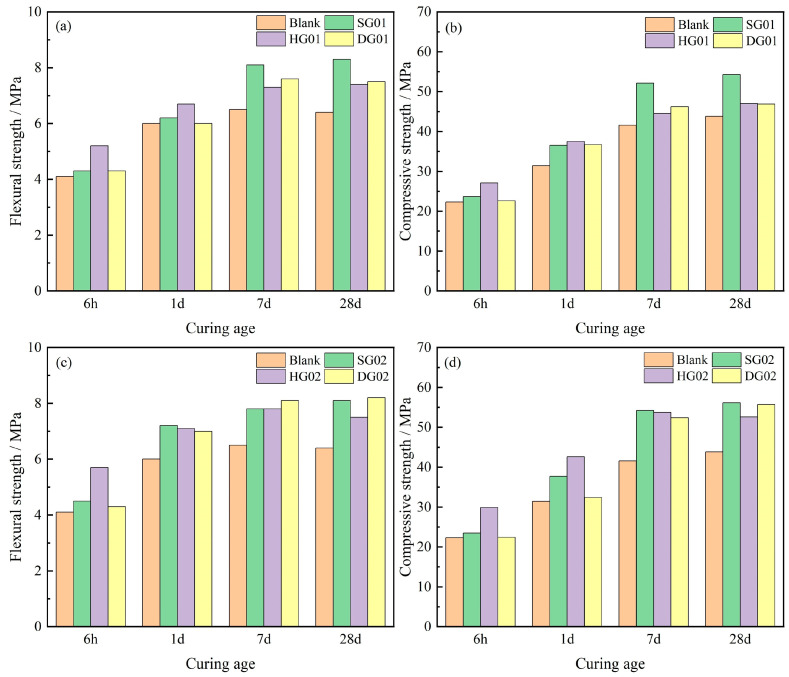
Mechanical strength of samples in different curing period. (**a**,**b**) are the flexural and compressive strengths of samples doped with 4% calcium sulfate, respectively; (**c**,**d**) are the flexural and compressive strengths of samples doped with 8% calcium sulfate, respectively.

Figure 3 shows the compressive and flexural strengths of SCRM samples at various ages. Figure 3a,b show the histograms of mechanical strengths of the experimental group with 4% calcium sulfate added versus the control group, and Figure 3c,d show the histograms of mechanical strengths of the experimental group doped with 8% calcium sulfate versus the control group. Figure 3a,b show that, after 6 h of hydration, the integration of HG greatly enhanced the mechanical characteristics of SCRM, with an increase in the flexural and compressive strengths of 26.83% and 21.52%, respectively, in comparison to the control group. With the addition of SG and DG, the experimental group’s strength was also increased; however, this improvement was not immediately apparent. The mechanical characteristics of SCRM were similarly most noticeably enhanced by HG at 1 day after hydration, with flexural and compressive strengths rising by 11.67% and 19.42%, respectively. With the inclusion of SG and DG, the experimental group’s strength also increased. After reaching 7 days of hydration, HG had a greater impact on SCRM strength. The experimental group that received SG addition exhibited the highest mechanical strength at 7 and 28 days among those that also received 4% calcium sulfate. For 7 days of hydration, the addition of 4% SG enhanced the flexural and compressive strengths of SCRM by 24.61% and 25.24%, respectively, and for 28 days of hydration, by 29.69% and 23.97%, respectively. At hydration levels up to 28 days, adding 4% HG and DG greatly increased the flexural strength while just slightly increasing the compressive strength.

The 6 h mechanical properties of SCRM are most noticeably improved by the addition of 8% HG, as shown by Figure 3c,d, where the 6 h flexural and compressive strengths are improved by 39.02% and 34.08%, respectively. However, the 28 d strength appeared to be inverted shrinkage, which may be due to the HG02 hydration rate being too fast, resulting in its not being involved in the early hydration of calcium sulfate by the rapid formation of the AFt package. The calcium sulfate comes into contact with water during post-conservation, absorbing water and dissolving it, leading to inverted shrinkage in the strength of the system. When SG02 is compared to the control group, the mechanical strength of hydration does not significantly rise for 6 h, but it does significantly increase for 1 day, and the strength continues to increase steadily over time. When the experimental group was maintained for up to 28 days with the addition of 8% calcium sulfate, DG02 had the highest flexural strength of 8.21 MPa, which is 28.12% higher than the control group, and SG02 had the highest compressive strength of 56.13 MPa, which is 28.8% higher.

### 3.3. Dry Shrinkage

**Figure 4:**
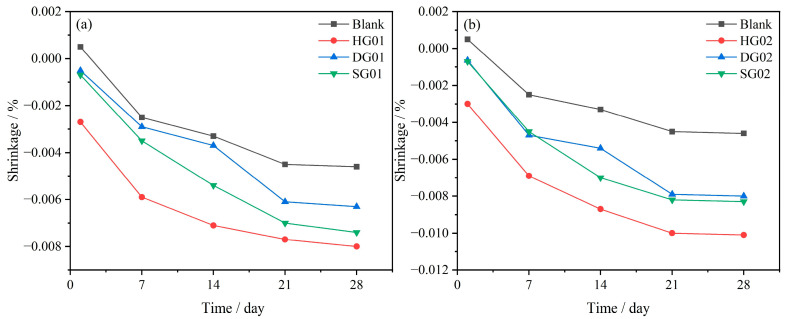
Dry shrinkage of self-compacting repair mortar (SCRM) prepared with three types of CaSO_4_. (**a**,**b**) Plots of dimensional changes for samples doped with 4% and 8% calcium sulfate, respectively.

Figure 4 shows the dry shrinkage of SCRM specimens at various ages. It is obvious that the control group displayed micro-expansion during the curing process, and the addition of three different types of calcium sulfate increased the visibility of SCRM’s expansion impact. The incorporation of 8% HG had the most noticeable impact on the specimens’ dimensional change, with 1 d and 28 d dry shrinkage less than −0.003% and −0.01%, respectively. This was related to the rapid dissolution of HG, which resulted in the formation of a significant amount of AFt in the early stage of hydration [28]. Combined with Figure 4a,b, it can be seen that SCRM exhibits greater expansion as the dosage of calcium sulfate is elevated from 4% to 8%.

Dry shrinkage, chemical shrinkage, and self-shrinkage are the three main types of cementitious material shrinkage [35]. The crystal growth theory and the expansion theory are the two hypotheses that can be used to explain the expansion of AFt [36]. The expansion phenomenon is thought to be caused by AFt crystals, which are formed when calcium sulfate and sulfoaluminate react, growing on the surface of cement particles and creating crystallization pressure. The swelling theory contends that the system swells as a result of AFt’s enormous surface area, which adsorbs water in the cementitious material. According to the swelling theory, the swelling capacity of the system will be improved, and it will be able to encapsulate more free water, which will reduce the amount of dry shrinkage. The experimental results demonstrate that as the gypsum content increases, the cementitious system produces more swelling at the early stage of hydration. The swelling impact of HG is more effective than that of SG and gypsum dihydrate in terms of the compensatory effect of gypsum. This results from the variance in gypsum’s solubility and rate of dissolution. Additionally, the form and growth pattern of the AFt have an impact on the compensating effect. Gypsum addition that is too high, however, might cause excessive swelling of SCRM cementitious materials and raise the early swelling rate. To completely utilize the shrinkage compensating effect of gypsum in practical applications without unnecessarily raising the early expansion rate, attention must be taken to limit the amount of gypsum employed.

### 3.4. XRD

**Figure 5:**
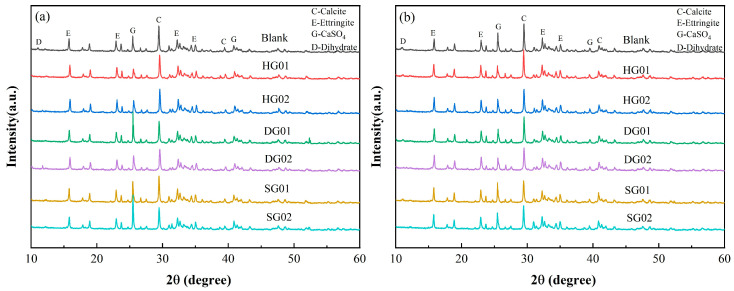
The X-ray diffraction pattern of samples at (**a**) 1 d and (**b**) 7 d.

The SCRM XRD patterns at 1 and 7 days are shown in Figure 5. Figure 5a demonstrates that the main hydration products of SCRM are AFt. Due to the fact that HG produces free Ca^2+^ and SO_4_^2−^, which are used to produce AFt during the rapid dissolving process, the addition of HG caused a decrease in the typical peak of CaSO_4_. Due to the slower dissolving rates of the two, the addition of SG and DG caused an increase in the characteristic peak of CaSO_4_, and some of the SG and DG did not participate in the 1 d hydration. A possible explanation for why DG01’s CaSO_4_ characteristic peak is greater than DG02’s is that DG02 contains more DG, which causes the dihydrate to dissolve and crystallize more quickly. This leaves less time for cement hydration to remove the binding water. Figure 5b shows that after 7 days of hydration, SG01 and SG02 still contain SG that is not involved in the reaction. This SG can also serve as a source of sulfate to encourage the full hydration of anhydrous calcium sulfoaluminate to form AFt in the later hydration of the SCRM, which is the main cause of the added SG’s higher mechanical strength at 28 days than that of the other experimental groups.

### 3.5. Heat Evolution of Binders

**Figure 6:**
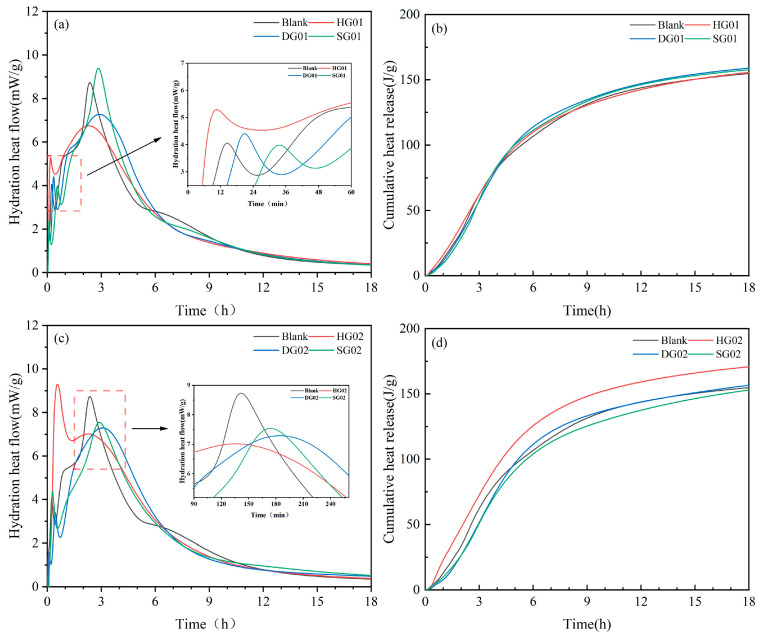
Early hydration heat of samples. (**a**,**c**) Heat of hydration curves for 4% and 8% calcium sulfate samples, respectively; (**b**,**d**) cumulative exothermic curves for 4% and 8% calcium sulfate samples, respectively.

Figure 6 displays the cumulative exothermic curves and early heat of hydration curves of several materials. These curves mostly reflect the exothermic hydration of cement clinker and gypsum in SCRM. Two exothermic peaks in SCRM can be seen by looking at the photos; these peaks produce a lot of heat when cementitious materials are mixed with water during the induction stage. From Figure 6a, it can be seen that the first exothermic peak appears first when HG is present, which indicates that HG plays a facilitating role in the early hydration of SCRM. Due to the high dissolution rate of α-hemihydrate, the early hydration of the ternary system containing α-hemihydrate rapidly forms AFt while releasing a large amount of heat, which can be seen in Figure 6c,d. It is clear that before hydration for one hour, HG02’s first exothermic peak and cumulative heat release are at their maximum levels. It is possible that the produced AFt wrapped around the C_3_S clinker and prevented it from coming into touch with water for the hydration process because the second exothermic peaks of the HG and the control group occurred at the same time, but the peak widths grew longer [34]. The first exothermic peak and the second exothermic peak of DG01 and DG02 were delayed and the peaks were smaller compared to the control, which showed that the addition of DG delayed the cementitious system’s early hydration process. The experimental group with the addition of 4% SG experienced the first exothermic peak later than the other two calcium sulfates, and the cumulative exothermic amount of 18 h was lowest of all experimental groups when SG doping was at 8%, indicating that SG could effectively slow the hydration rate.

Overall, the HG facilitated the conversion of anhydrous calcium sulfoaluminate to Aft and accelerated the hydration rate by increasing the ionized Ca^2+^ and SO_4_^2−^ in the early hydration process. The system’s hydration was slowed considerably by both DG and SG. Due to the creation of the amorphous phase C-A-S-H gel, the second exothermic peak width of DG is the largest among them.

### 3.6. Porosity

An essential component of cementitious materials’ microstructure, the internal pore structure, has a significant impact on their mechanical, durability, and shrinkage characteristics. In general, the pores in cementitious materials can be categorized into four types: <20 nm harmless pores, 20–50 nm mesopores, 50–200 nm harmful pores, and >200 nm larger harmful pores [37].

**Figure 7:**
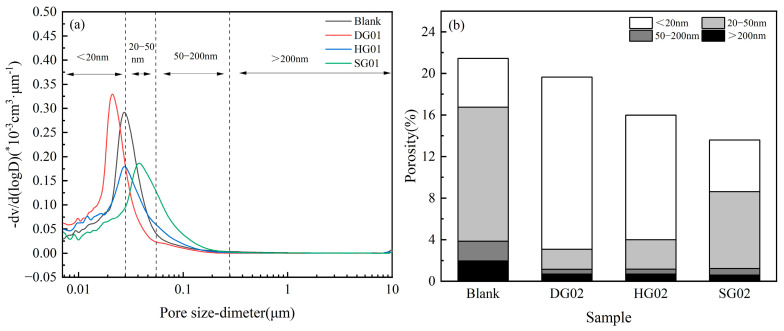
(**a**) Pore size distribution and (**b**) porosity of samples (14 d).

Following 14 days of hydration, the SCRM’s porosity and pore size distribution are depicted in Figure 7a and Figure 7b, respectively. Observing the images, it can be clearly seen that compared with the control group, the pore size distribution curves of the experimental group doped with 8% calcium sulfate were all shifted to the left, which indicates that the addition of the three different types of calcium sulfate makes the pores of SCRM smaller, promotes the transformation of harmful pores to harmless pores, and the structure becomes denser. After the cementitious materials had been hydrated for 14 days, the total porosity and the contents of different types of pores in the samples from the experimental group that had been doped with 8% calcium sulfate were examined in order to better understand the impact of calcium sulfate on the porosity of SCRM. The percentage of the overall porosity and the different kinds of pores are shown in Figure 7b. The samples’ total porosity can be shown to be decreasing from left to right, which suggests that the three different forms of calcium sulfate have the ability to convert dangerous SCRM pores into safe pores. The presence of calcium sulfate, the rise in Ca^2+^ and SO_4_^2−^ concentrations during the hydration process, which encourages the production of AFt, and the filling of pores by AFt, which reduces total porosity, are the causes of this. As demonstrated by Reactions (1) and (3), the amount of Ca^2+^ and SO_4_^2−^ ionized in the early stages of DG is smaller than that of HG. As a result, less AH_3_ is produced in the early stages of the former than the latter, which causes the experimental group of HG02 to produce more C_2_ASH_8_. Typically, C_2_ASH_8_ is present as a gel that homogenizes the slurry and plugs the open pore spaces. It was discovered that HG02 has less overall porosity than DH02 does. Due to the presence of SG, SG02 has the lowest overall porosity of the group. The reaction kinetics of creating AFt are affected by the sluggish release of Ca^2+^ and SO_4_^2−^, which leads to the formation of AFt with a smaller size. This smaller AFt is better able to fill the pores and, as a result, results in a denser substrate.

## 4. Conclusions

Using the previous data in conjunction, it can be concluded that HG speeds up the formation of Aft, early mechanical strength develops quickly, and the pore structure of the system can be improved. However, the loss of mobility of SCRM increases, and the larger expansion increases the material’s risk of expansion and cracking in the future. The system’s rate of hydration is significantly slowed down by the addition of DG, although there is little immediate gain in mechanical strength and the initial mobility decreases. With the lowest total porosity and highest mechanical strength in the later stage, the effects of SG on the SCRM exhibited the best improvement in workability.

This research focuses on the impact of three different forms of calcium sulfate inclusion on the hydration and performance of SCRM in an effort to enhance its working performance. The following are the primary conclusions:

- The condensation time of SCRM was delayed to varying degrees by the addition of all three calcium sulfates. In comparison to hemihydrate and dihydrate gypsum, the SCRM produced with the addition of anhydrite demonstrated the least fluidity loss and the longest setting time. The best kind of calcium sulfate to use for making SCRM is anhydrite.
- A ternary cementitious system with an appropriate amount of calcium sulfate incorporated can effectively improve the mechanical strength and stability of mortar. Compared with the control group, the SCRM (HG02) doped with hemihydrate gypsum showed the greatest increase in the early 6 h flexural and compressive strengths, which were enhanced by 39.02% and 34.08%, respectively. The mechanical strength of SCRM increased as the calcium sulfate dose increased from 4% to 8%. The addition of calcium sulfate enhanced the expansion characteristics of the cementitious system dominated by sulfoaluminate cement. The compensatory swelling effect of the three gypsums was hemihydrate gypsum > anhydrite > dihydrate gypsum.
- When calcium sulfate was added to the cementitious system, varied calcium sulfate types, solubilities, and rates of dissolution resulted in different levels of Ca^2+^ and SO_4_^2−^ throughout the system’s hydration process, which in turn altered the hydration of cement and the production of AFt. The addition of hemihydrate gypsum accelerates the hydration process compared to the other two calcium sulfates. The hydration process was slowed down by the addition of anhydrite and dihydrate gypsum.
- The addition of calcium sulfate led to a denser microstructure and decreased porosity in SCRM because anhydrite calcium sulfoaluminate had been more thoroughly hydrated. The inclusion of anhydrite resulted in a superior crystalline structure of AFt from hydration compared to hemihydrate and dihydrate gypsum, making the structure denser and reducing total porosity.

## Figures and Tables

**Table 1:** Chemical compositions of cements and calcium sulfates used in this study (wt%).

Materials	CaO	SiO_2_	Al_2_O_3_	Fe_2_O_3_	SO_3_	MgO	TiO_2_	LOI
OPC	57.67	20.69	6.3	5.74	2.31	1.9	0.38	3.91
SAC	43.34	10.04	17.2	2.41	12.61	2.19	0.86	9.93
HG	37.18	0.21	0.09	0.04	55.64	0.04	0.02	6.44
SG	38.17	1.37	0.07	0.02	52.77	4.09	0.05	2.81
DG	22.80	15.49	5.34	1.37	31.99	3.87	0.21	16.21

**Table 2:** Composition of self-compacting repair mortar (SCRM) mix formulations (wt%).

No.	HG	DG	SG	SAC	OPC
Blank	0	0	0	75	25
HG01	4	0	0	72	24
HG02	8	0	0	69	23
DG01	0	4	0	72	24
DG02	0	8	0	69	23
SG01	0	0	4	72	24
SG02	0	0	8	69	23

**Table 3:** Setting time and flow value of self-compacting repair mortar (SCRM).

No.	Setting Time/min		Flow Value/mm		
	Initial	Final	Initial	20 min	Loss
Blank	30	40	308	273	35
HG01	49	59	291	230	61
HG02	47	53	290	225	65
DG01	42	60	300	251	49
DG02	40	62	299	255	44
SG01	67	102	300	267	33
SG02	71	114	301	271	30

## Data Availability

Not applicable.

## References

[1] He J., Bu X., Bai W., Zheng W., Gao Q., Wang Y. (2020). Preparation and properties of self-compacting alkali-activated slag repair mortar—ScienceDirect. Constr. Build. Mater..

[2] Wojnowski D., Francke B., Garbacz A. (2020). Influence of Lowered Temperature on Efficiency of Concrete Repair with Polymer-Cement Repair Mortars. Materials.

[3] Felekoğlu B., Türkel S., Altuntaş Y. (2007). Effects of steel fiber reinforcement on surface wear resistance of self-compacting repair mortars. Cem. Concr. Compos..

[4] FelekoğLu B., Tosun K., Baradan B., Altun A.N., Uyulgan B.R. (2006). The effect of fly ash and limestone fillers on the viscosity and compressive strength of self-compacting repair mortars. Cem. Concr. Res..

[5] Isebaert A., Parys L.V., Cnudde V. (2014). Composition and compatibility requirements of mineral repair mortars for stone—A review. Constr. Build. Mater..

[6] Zhang J., Guan X., Wang X., Ma X., Jin B. (2020). Microstructure and Properties of Sulfoaluminate Cement-Based Grouting Materials: Effect of Calcium Sulfate Variety. Adv. Mater. Sci. Eng..

[7] Elakneswaran Y., Noguchi N., Matumoto K., Morinaga Y., Nawa T. (2019). Characteristics of Ferrite-Rich Portland Cement: Comparison With Ordinary Portland Cement. Front. Mater..

[8] Tan H., Zhang X., He X., Guo Y., Deng X., Su Y., Yang J., Wang Y. (2018). Utilization of lithium slag by wet-grinding process to improve the early strength of sulphoaluminate cement paste. J. Clean. Prod..

[9] Quillin K. (2001). Performance of belite–sulfoaluminate cements. Cem. Concr. Res..

[10] Aranda M.A.G., De la Torre A.G. (2013). Sulfoaluminate Cement.

[11] Lin R., Yang L., Pan G., Sun Z., Li J. (2021). Properties of composite cement-sodium silicate grout mixed with sulphoaluminate cement and slag powder in flowing water. Constr. Build. Mater..

[12] Zhang Y., Li T., Feng W., Xiong Z., Zhang G. (2020). Effects of temperature on performances and hydration process of sulphoaluminate cement-based dual liquid grouting material and its mechanisms. J. Therm. Anal. Calorim..

[13] Nie S., Zhang Q., Lan M., Zhou J., Xu M., Li H., Wang J. (2023). Fundamental design of low-carbon ordinary Portland cement-calcium sulfoaluminate clinker-anhydrite blended system. Cem. Concr. Compos..

[14] Sun G., Wang Z., Yu C., Qian X., Chen R., Zhou X., Weng Y., Song Y., Ruan S. (2023). Properties and microstructures of 3D printable sulphoaluminate cement concrete containing industrial by-products and nano clay. J. Build. Eng..

[15] Guo C., Wang R. (2023). Using sulphoaluminate cement and calcium sulfate to modify the physical–chemical properties of Portland cement mortar for mechanized construction. Constr. Build. Mater..

[16] Gastaldi D., Paul G., Marchese L., Irico S., Boccaleri E., Mutke S., Buzzi L., Canonico F. (2016). Hydration products in sulfoaluminate cements: Evaluation of amorphous phases by XRD/solid-state NMR. Cem. Concr. Res..

[17] Tang R., Sun D., Wang Z., Wang Z., Cui S., Ma W., Lan M. (2023). Synergistic Effect and Mechanism of Nano-C-S-H Seed and Calcium Sulfoaluminate Cement on the Early Mechanical Properties of Portland Cement. Materials.

[18] Ji G., Ali H.A., Sun K., Xuan D., Peng X., Li J. (2023). Volume Deformation and Hydration Behavior of Ordinary Portland Cement/Calcium Sulfoaluminate Cement Blends. Materials.

[19] Hongyin L., Jiandang D., Xin Y. (2015). Study on the performance of modified repair materials compounded with ordinary silicate cement and sulfoaluminate cement. Archit. Technol..

[20] Zhou J., Chen L., Zheng K., Prateek G., He F., Liu Z., Yuan Q. (2023). Effect of elevated Al concentration on early-age hydration of Portland cement. Cem. Concr. Compos..

[21] Palou M., Majling J., Dovál M., Kozanková J., Mojumdar S.C. (2005). Formation and stability of crystallohydrates in the non-equilibrium system during hydration of sab cements. Ceram. Silik..

[22] Saeidpour, Mahsa, Matschei, Thomas, Scrivener, Karen L., Wadso, Lars, Baquerizo, Luis G. (2015). Hydration states of AFm cement phases. Cem. Concr. Res..

[23] Gastaldi D., Canonico F., Capelli L., Bianchi M., Valenti G.L. Hydraulic Behaviour of Calcium Sulfoaluminate Cement Alone and in Mixture with Portland Cement. Proceedings of the 13th International Congress on the Chemistry of Cement.

[24] Winnefeld F., Barlag S. (2010). Calorimetric and thermogravimetric study on the influence of calcium sulfate on the hydration of ye’elimite. J. Therm. Anal. Calorim..

[25] Telesca A., Marroccoli M., Winnefeld F. (2019). Synthesis and characterisation of calcium sulfoaluminate cements produced by different chemical gypsums. Adv. Cem. Res..

[26] Allevi S., Marchi M., Scotti F., Bertini S., Cosentino C. (2015). Hydration of calcium sulphoaluminate clinker with additions of different calcium sulphate sources. Mater. Struct..

[27] García-Maté M., De la Torre A.G., León-Reina L., Losilla E.R., Aranda M.A.G., Santacruz I. (2015). Effect of calcium sulfate source on the hydration of calcium sulfoaluminate eco-cement. Cem. Concr. Compos..

[28] Andrade Neto J.S., de Matos P.R., De la Torre A.G., Campos C.E.M., Gleize P.J.P., Monteiro P.J.M., Kirchheim A.P. (2022). The role of sodium and sulfate sources on the rheology and hydration of C3A polymorphs. Cem. Concr. Res..

[29] (2016). Repair Mortar.

[30] (2001). Test Methods for Water Requirement of Normal Consistency, Setting Time and Soundness of the Portland Cement.

[31] (2005). Cement-Based Self-Leveling Paddles for Floors.

[32] (1999). Method of Testing Cements-Determination of Strength.

[33] Carrasco L.F. (2015). Conduction calorimetric studies of ternary binders based on Portland cement, calcium aluminate cement and calcium sulphate. J. Therm. Anal. Calorim..

[34] Andrade Neto J.d.S., De la Torre A.G., Kirchheim A.P. (2021). Effects of sulfates on the hydration of Portland cement—A review. Constr. Build. Mater..

[35] Liu L., Wang X., Chen H., Wan C. (2016). Microstructure-based modelling of drying shrinkage and microcracking of cement paste at high relative humidity—ScienceDirect. Constr. Build. Mater..

[36] Evju C., Hansen S. (2005). The kinetics of ettringite formation and dilatation in a blended cement with β-hemihydrate and anhydrite as calcium sulfate. Cem. Concr. Res..

[37] Wang Q., Wang J., Lu C.X., Liu B.W., Li C.Z. (2015). Influence of graphene oxide additions on the microstructure and mechanical strength of cement. Xinxing Tan Cailiao/New Carbon Mater..

